# Association of Maternal Preferred Language with Breastfeeding Attitudes, Intentions, and Knowledge

**DOI:** 10.34763/jmotherandchild.20232701.d-23-00026

**Published:** 2023-11-22

**Authors:** Lincoln Ferguson, Alexandra Chervonsky, Joshua Fogel, Allan J. Jacobs

**Affiliations:** Department of Obstetrics and Gynecology, New York Presbyterian Brooklyn Methodist Hospital, Brooklyn, New York, USA; Department of Obstetrics and Gynecology, South Brooklyn Health, Brooklyn, New York, USA; New York Institute of Technology College of Osteopathic Medicine, Old Westbury, New York, USA; Department of Business Management, Brooklyn College, Brooklyn, New York, USA.; Department of Obstetrics and Gynecology, Downstate Medical Center, Brooklyn, New York, USA

**Keywords:** breastfeeding, theory of planned behaviour, ethnicity, lactation, lactation education

## Abstract

**Introduction:**

Assessing intentions, attitudes, and knowledge about breastfeeding among different language groups is important because the languages reflect cultural differences. We compared attitudes, subjective norms, perceived behavioural control, intentions, and knowledge of breastfeeding among mothers with the five most common preferred languages spoken at a New York City hospital.

**Materials and methods:**

This cross-sectional study surveyed women (n = 448) in the prenatal clinic and the post-partum unit of a New York City hospital. The survey questions were about breastfeeding attitudes, subjective norms, perceived behavioural control, and intentions, based on the Theory of Planned Behavior. We also administered the Iowa Infant Feeding and Attitude Scale and measured the knowledge of the mothers about breastfeeding. The preferred language spoken by the mother was the main predictor variable. English, Russian, Spanish, Urdu, and Uzbek were the languages studied.

**Results:**

Multivariate linear regression analyses showed that Russian (B = 2.24, SE = 1.09, p = 0.04), Urdu (B = 2.90, SE = 1.45, p = 0.046), and Uzbek (B = 4.21, SE = 1.35, p = 0.002) speakers all had significantly more positive attitudes toward breastfeeding than did English speakers. Spanish and English language speakers did not differ from each other in their attitudes towards breastfeeding. The language groups did not differ significantly for subjective norms, perceived behavioural control, intention to breastfeed, the Iowa Infant Feeding and Attitude Scale, nor in knowledge regarding breastfeeding.

**Conclusions:**

Urdu, Uzbek, and Russian speakers had significantly more positive attitudes towards breastfeeding than did English speakers. To the extent that preferred language is a proxy for culture, clinicians can use this parameter as a basis for directing approaches toward lactation education.

## Introduction

The benefits of breastfeeding are among the factors that motivate women to decide whether to initiate breastfeeding for their infants and why they choose to continue to do so. We try to unpack some cultural differences that determine breastfeeding choices using the Theory of Planned Behavior (TPB) [1] as a framework for analysis. The TPB is extensively used worldwide to determine and understand why people make specific choices [2,3]. The TPB framework classifies the following as potentially influencing personal choices: personal attitudes, subjective norms (a term of art meaning perceived social pressure), and perceived behavioural control (perceived ability to perform a behaviour as regulated by tangible external constraints). These three factors influence actors’ intentions. Their intentions, in turn, influence behaviour [1].

The American College of Obstetricians and Gynecologists (ACOG) regards breastfeeding education as important for infant health and recommends providing patients with information and guidance [4]. A meta-analysis reports that educational interventions about breastfeeding were associated with increased exclusive breastfeeding during the first week, and at four to six weeks postpartum [5]. A study of women attending a clinic in the United States found that Hispanic women who preferred to communicate in English knew more about breastfeeding than did Hispanic women who preferred to communicate in Spanish [6].

The TPB had previously been applied to breastfeeding. All three TPB parameters of attitudes, subjective norms, and perceived behavioural control were positively associated with breastfeeding intentions [7,8] and breastfeeding persistence at six weeks postpartum [9]. A study comparing different racial/ethnic groups found that attitudes and subjective norms, but not perceived behavioural control, were positively associated with exclusive breastfeeding among white and Black groups [10]. Among Hispanics, though, the opposite pattern occurred. Perceived behavioural control, but not attitudes and subjective norms, was positively associated with exclusive breastfeeding [10].

The Iowa Infant Feeding and Attitudes Scale (IIFAS)^9^ is widely used to study attitudes and practices regarding infant feeding [11,12,13]. In a Spanish study using language as a proxy for culture, primary Chinese-speaking mothers displayed lower IIFAS scores than primarily Spanish-speaking mothers [14]. A study from three nations comparing British, Russian, and Chinese mothers found that Russian and Chinese mothers had more neutral attitudes while British mothers had more positive attitudes towards breastfeeding [13].

We are unaware of any studies about breastfeeding intentions, attitudes, and knowledge among those primarily speaking Urdu or Uzbek. The literature applying the TPB to breastfeeding intentions either focuses on single language speaking groups [15] or compares different race/ethnicities [10]. The literature on breastfeeding attitudes compares some language groups but does not evaluate Urdu and Uzbek speakers. Neither does the literature on breastfeeding attitudes compare Spanish and Russian speakers living in the US. The literature on breastfeeding knowledge compares English and Spanish speakers living in the US but does not compare Russian, Urdu, and Uzbek speakers living in the US. Assessing intentions, attitudes, and knowledge of breastfeeding among different language groups is important because of cultural differences among these groups. Understanding these differences may lead to creation of better-informed decisions regarding breastfeeding. The setting for this study is a hospital serving indigent and working-class people in a large US city with many immigrant parturients; this hospital provides a useful setting to study breastfeeding intentions, attitudes, and knowledge among multiple language groups. In our study, we compare English, Russian, Spanish, Urdu, Uzbek, and other language speakers.

## Materials and methods

### Setting

This cross-sectional study surveyed women in the prenatal clinic and the postpartum inpatient service of a New York City public hospital. Pregnant patients and those who had delivered a live infant within the previous 72 hours were included. Patients were surveyed anonymously from March 2021 through June 2022. Patients had the choice of completing the survey in English, Arabic, Russian, Spanish, Urdu, or Uzbek, languages that correspond to the most frequent primary languages spoken by patients in the hospital. Patients whose primary language was not one of these languages completed the survey independently without assistance in one of the above languages in which they were literate, usually English. Urdu is the predominant language of Pakistan. Uzbek is the primary language of most Muslims native to Uzbekistan; few of the many immigrants from Uzbekistan who speak Russian or a Persian dialect receive care at this hospital. All patients provided informed consent. The study was ethically conducted and received institutional review board approval.

## Variables

### Main predictor variable

Preferred language spoken was the main predictor variable. Patients were categorized as speakers preferring English, Russian, Spanish, Urdu, Uzbek, or other. Because there were only seven Arabic-preferred language responses, these were included within the ‘other’ category.

### Covariates

Demographic covariates included age (years), self-identified race/ethnicity (white, African-American, Hispanic, Asian, other), whether born in the US, how many years lived in the US, and educational level (elementary or less, some high school, completed high school, associate degree, bachelor's degree, and master's/doctoral degree). Pregnancy variables included gravidity, parity, number of miscarriages/abortions, and current pregnancy status (currently pregnant or recent delivery of live infant). Breastfeeding variables were whether the patient had previously breastfed a child and whether a close relative (mother, sister, sister-in-law) had breastfed a child.

### Outcome variables

The TPB postulates that variables which influence behaviour can be classified as attitudes, subjective norms, perceived behavioural control, and intentions [1]. We created scales to assess each of these categories as applied to breastfeeding, using the published framework for creating TPB variables [16]. All scales measured responses using a Likert-style approach ranging from 1 to 7.

The attitudes scale had four items. All items began with, ‘Breastfeeding my child is:’ and the four item responses ranged from (1) harmful = 1 to beneficial = 7, (2) bad = 1 to good = 7, (3) unpleasant = 1 to pleasant = 7, and (4) worthless = 1 to useful = 7.

The subjective norms scale had three items. These items were (1) ‘Most people who are important to me think that I [should not = 1 or should = 7] breastfeed my child’; (2) ‘It is expected of me that I breastfeed my child’ ranging from strongly disagree = 1 to strongly agree = 7; and (3) ‘Most people who are important to me want me to breastfeed my child’ ranging from strongly disagree = 1 to strongly agree = 7. The perceived behavioural control scale had three items: (1) ‘I am confident that if I wanted to, I could breastfeed my child’ ranging from strongly disagree = 1 to strongly agree = 7; (2) ‘For me to breastfeed my child is’ ranging from difficult = 2 to easy = 7; and (3) ‘Whether I make a decision about breastfeeding my child is entirely up to me’ ranging from strongly disagree =1 to strongly agree = 7.

The intentions scale had three items: (1) ‘I expect to breastfeed my child’; (2) ‘I want to breastfeed my child’; and (3) ‘I intend to breastfeed my child’ all ranging from strongly disagree = 1to strongly agree = 7. Items in each scale were added together for a total score indicating a greater score for that scale. Cronbach alpha reliability in our sample was attitudes = 0.8, subjective norms = 0.6, behavioural control = 0.6 and intentions = 0.9, demonstrating adequate internal consistency of these items.

The IIFAS has 17 items [11]. Items were measured on a Likert-style scale ranging from strongly disagree = 1 to strongly agree = 7. Nine of the items were reverse coded. A sample item is, ‘Breast milk is more easily digested than formula’. Cronbach alpha reliability in our sample was 0.6, demonstrating adequate internal consistency.

Breastfeeding knowledge was measured using 13 of the 14 items from a previously published scale [17]. We removed one item whose answer, published at the time as correct, is not consistent with current medical consensus. Answer choices were either true, false, or unsure. ‘True’ was considered correct for seven questions, and ‘false’ was considered correct for six questions. A sample ‘true correct’ item is, ‘Infants who have breastfed have a reduced risk of getting diabetes.’ We created two measures consisting of the total number answering ‘correct’ to measure correct knowledge and total number choosing ‘unsure’ to measure level of unsureness for the knowledge questions. Kuder-Richardson 20 reliability in our sample was knowledge correct = 0.7 and knowledge unsure = 0.7, demonstrating adequate internal consistency.

### Statistical analyses

Mean and standard deviation were used to describe continuous variables. Frequency and percentage were used to describe categorical variables. Continuous variables were compared using analysis of variance. Categorical variables were compared using the Pearson chi-square test. The least significant difference (LSD) post-hoc test compared the language categories when there was an overall statistical significance for the continuous outcome variables. All covariates significantly differing (*p* < 0.05) or approaching significance (*p* < 0.052) between the language groups were included as predictor variables in the multivariate linear regression analysis for outcome variables that significantly differed in the univariate analyses. Variables with a skewed distribution with a value of zero that were logarithmically transformed had the value of one added due to logarithmic transformations being unable to be performed for a value of zero. Internal consistency was calculated using Cronbach alpha for the Likert-style scale outcomes and the Kuder-Richardson 20 for the dichotomous ‘total knowledge correct’ and ‘total knowledge unsure’ items. IBM SPSS Statistics version 28 was used for all analyses [18]. All *p*-values were two-tailed with alpha <0.05.

Imputation of the mean of the participant was done for the intentions, attitudes, subjective norms, and behavioural control scales when there was one missing response. Imputation of the mean of the participant was done for the IIFAS when there were three or fewer missing responses. Imputation of ‘unsure’ was done for the knowledge scale when there were two or fewer missing responses.

## Results

The patients in each language group were English (*n* = 136, 30.4%), Russian (*n* = 58, 12.9%), Spanish (*n* = 104, 23.2%), Urdu (*n* = 42, 9.4%), Uzbek (*n* = 46, 10.3%), and other (*n* = 62, 13.8%). Table 1 shows the sample characteristics and comparisons among the language groups. US birth (*p* < 0.001) and length of US residence (*p* < 0.001) significantly differed. Uzbek speakers had the lowest percentage of each of these responses. Education levels significantly differed among language groups (*p* < 0.001). Russian speakers had the greatest percentage of individuals with graduate degrees. Gravidity (*p* = 0.001) and parity (*p* < 0.001) significantly differed; Uzbek speakers had the greatest means of each. Uzbek speakers also had the greatest percentage to have breastfed a previous child (*p* < 0.001) and for those who recently gave birth (*p* < 0.001). Mean age differences approached significance (*p* = 0.051). The age mean ranged from 29.7 years for English speakers to 32.7 years for Russian speakers. Having a close relative who breastfed a child also approached significance (*p* = 0.050) with the greatest percentage of breastfeeding relatives among Russian speakers.

**Table 1.** Comparisons of Sample Characteristics for the 448 Participants.

**Variable**	**English M (SD) or # (%) (n=136)**	**Russian M (SD) or # (%) (n=58)**	**Spanish M (SD) or # (%) (n=104)**	**Urdu M (SD) or # (%) (n=42)**	**Uzbek M (SD) or # (%) (n=46)**	**Other M (SD) or # (%) (n=62)**	**p-value**
Age (years) [mean]	29.7 (5.76)	32.4 (5.25)	30.1 (6.69)	29.6 (4.66)	29.3 (4.88)	30.0 (4.82)	0.051
Race/ethnicity							<0.001
White	30 (22.2)	25 (44.6)	1 (1.0)	0 (0.0)	11 (25.6)	36 (60.0)	
African-American	38 (28.1)	3 (5.4)	1 (1.0)	0 (0.0)	0 (0.0)	4 (6.7)	
Hispanic	32 (23.7)	1 (1.8)	81 (81.8)	0 (0.0)	0 (0.0)	1 (1.7)	
Asian	25 (18.5)	11 (19.6)	0 (0.0)	37 (88.1)	25 (58.1)	13 (21.7)	
Other	10 (7.4)	16 (28.6)	16 (16.2)	5 (11.9)	7 (16.3)	6 (10.0)	
Born in USA (yes)	79 (59.4)	5 (8.6)	4 (3.9)	1 (2.4)	0 (0.0)	3 (4.8)	<0.001
Years lived in USA [mean]	20.9 (11.19)	7.2 (7.16)	9.7 (7.34)	12.5 (8.00)	4.7 (3.45)	6.0 (7.44)	<0.001
Education							<0.001
Elementary	2 (1.5)	0 (0.0)	32 (33.3)	2 (4.9)	0 (0.0)	2 (3.3)	
Some HS	19 (14.5)	10 (17.5)	32 (33.3)	6 (14.6)	10 (22.2)	6 (9.8)	
Completed HS	38 (19.0)	9 (15.8)	26 (27.1)	11 (26.8)	16 (35.6)	12 (19.7)	
Associate	27 (20.6)	7 (12.3)	2 (2.1)	5 (12.2)	14 (31.1)	11 (18.0)	
Bachelor	24 (18.3)	21 (36.8)	2 (2.1)	13 (31.7)	5 (11.1)	20 (32.8)	
Master/Doctoral	21 (16.0)	10 (17.5)	2 (2.1)	4 (9.8)	0 (0.0)	10 (16.4)	
Gravidity [mean]	2.7 (1.69)	3.1 (3.11)	2.9 (1.80)	2.5 (1.45)	3.4 (1.74)	2.1 (1.35)	0.001
Parity [mean]	2.1 (1.51)	2.0 (0.97)	2.3 (1.48)	2.1 (1.30)	2.9 (1.20)	1.7 (1.24)	<0.001
Number miscarriages/abortions [mean]	0.7 (1.02)	0.8 (1.16)	0.5 (0.73)	0.5 (0.71)	0.8 (1.14)	0.4 (0.64)	0.27
Recently had baby (yes)	67 (49.6)	32 (57.1)	48 (46.6)	17 (41.5)	36 (78.3)	43 (69.4)	<0.001
Breastfed previous child (yes)	69 (53.5)	36 (69.2)	67 (65.0)	21 (53.8)	40 (90.9)	25 (43.9)	<0.001
Close relative breastfed child (yes)	108 (81.2)	45 (83.3)	71 (70.3)	31 (75.6)	28 (60.9)	47 (77.0)	0.050

*Note*: M=mean, SD=standard deviation. USA=United States of America, HS=high school. Sample size can slightly vary due to omissions from participants. Gravidity and number miscarriages/abortions had skewed distributions and were logarithmic transformed for analyses for the reporting of the p-value. The mean and standard deviation values shown are for non-transformed values. Race/ethnicity and education had certain expected cell sizes <5 but the Fisher's exact test could not be performed and instead were analyzed with the Pearson chi-square test.

Table 2 shows univariate comparisons among the language groups for the outcomes. Mean attitude scores significantly differed among language groups. LSD post-hoc tests showed greater mean values indicative for more positive attitudes for Russian versus English speakers (*p* = 0.02), Spanish versus English speakers (*p* = 0.003), Urdu versus English speakers (*p* = 0.01), and Uzbek versus English speakers (*p* < 0.001). Mean subjective norm scores also significantly differed among language groups. LSD post-hoc tests showed mean values indicative of more positive subjective norms for Spanish versus English speakers (*p* < 0.001), Urdu versus English speakers (*p* = 0.01), and Spanish versus Russian speakers (*p* = 0.003). Mean perceived behavioural control scores differed significantly. LSD post-hoc tests showed greater mean values indicative for more positively perceived behavioural control for Spanish versus English speakers (*p* = 0.01), Uzbek versus English speakers (*p* = 0.002), Spanish versus Russian speakers (*p* = 0.046), Uzbek versus Russian speakers (*p* = 0.01), and Uzbek versus Urdu speakers (*p* = 0.04). The mean scores for intention to breastfeed, the IIFAS, and knowledge regarding breastfeeding did not significantly differ among the language groups.

**Table 2.** Univariate Comparisons for Outcomes.

**Variable**	**English M (SD) (n=136)**	**Russian M (SD) (n=58)**	**Spanish M (SD) (n=104)**	**Urdu M (SD) (n=42)**	**Uzbek M (SD) (n=46)**	**Other M (SD) (n=62)**	**p-value**	**Post-hoc**
Attitudes	23.2 (6.37)	25.3 (4.82)	25.6 (4.33)	26.1 (3.10)	27.0 (4.07)	25.2 (5.24)	0.001	Russian>English (p=0.02)
								Spanish>English (p=0.003)
								Urdu>English (p=0.01)
								Uzbek>English (p<0.001)
								Other>English (p=0.02)
Subjective norms	16.6 (4.61)	17.0 (4.77)	19.0 (3.39)	18.6 (3.03)	17.6 (4.52)	18.3 (2.92)	<0.001	Spanish>English (p<0.001)
								Urdu>English (p=0.01)
								Other>English (p=0.01)
								Spanish>Russian (p=0.003)
Behavioral control	17.9 (3.14)	17.9 (3.54)	19.0 (2.67)	18.2 (3.56)	19.7 (2.59)	18.0 (3.35)	0.01	Spanish>English (p=0.01)
								Uzbek>English (p=0.002)
								Spanish>Russian (p=0.046)
								Uzbek>Russian (p=0.01)
								Uzbek>Urdu (p=0.04)
								Uzbek>Other (p=0.01)
Intentions	17.9 (4.99)	18.8 (4.39)	19.3 (3.41)	18.6 (4.07)	17.5 (5.44)	19.1 (3.43)	0.11	
IIFAS	61.3 (8.25)	60.0 (6.45)	59.8 (5.74)	60.1 (6.23)	58.8 (6.61)	61.5 (7.26)	0.17	
Knowledge correct	6.6 (3.04)	6.1 (2.96)	6.1 (2.17)	7.0 (2.64)	6.5 (2.48)	6.1 (2.74)	0.31	
Knowledge unsure	4.4 (3.17)	4.8 (3.10)	4.2 (2.76)	3.8 (3.01)	3.6 (2.44)	4.8 (2.55)	0.16	

*Note*: M=mean, SD=standard deviation, IIFAS=Iowa Infant Feeding and Attitudes Scale. Sample size can slightly vary due to omissions from participants.

Table 3 summarizes multivariate linear regression analyses. For language, Russian, Urdu, and Uzbek speakers all had significantly more positive attitudes toward breastfeeding than did preferred English speakers. On the other hand, there were no significantly different language differences regarding subjective norms or perceived behavioural control variables. African-Americans had significantly greater positive subjective norms and approached significance for positive perceived behavioural control (*p* = 0.050) as compared to white subjects. Increased gravidity was significantly associated with decreased positive attitudes and decreased positive subjective norms. Increased parity was associated with increased positive subjective norms. Having breastfed a previous child was significantly associated with increased positive perceived behavioural control. A woman's having a close relative who breastfed a child was associated significantly with increased positive attitudes toward breastfeeding, subjective norms, and perceived behavioural control.

**Table 3.** Multivariate Linear Regression Analyses for Attitudes, Subjective Norms, and Behavioral Control.

**Variable**	**Attitudes B (SE)**	**p-value**	**Subjective Norms B (SE)**	**p-value**	**Behavioral Control B (SE)**	**p-value**
Language						
English	Reference		Reference		Reference	
Russian	2.24 (1.09)	0.04	0.98 (0.77)	0.21	−0.87 (0.63)	0.17
Spanish	2.19 (1.27)	0.09	1.34 (0.86)	0.12	−0.08 (0.70)	0.91
Urdu	2.90 (1.45)	0.046	1.21 (0.92)	0.19	0.14 (0.77)	0.86
Uzbek	4.21 (1.35)	0.002	0.88 (0.92)	0.34	0.22 (0.75)	0.77
Other	1.49 (1.07)	0.16	1.24 (0.75)	0.10	−1.08 (0.61)	0.08
Age (years)	0.07 (0.07)	0.33	−0.08 (0.04)	0.07	0.02 (0.04)	0.61
Race/ethnicity						
White	Reference		Reference		Reference	
African-American	0.17 (1.16)	0.32	3.09 (0.83)	<0.001	1.32 (0.67)	0.05
Hispanic	−0.55 (1.20)	0.65	1.06 (0.83)	0.20	0.05 (0.68)	0.94
Asian	0.17 (0.97)	0.86	0.87 (0.67)	0.19	−0.15 (0.54)	0.79
Other	0.25 (1.05)	0.81	1.24 (0.75)	0.81	0.05 (0.60)	0.93
Born in USA (yes)	−1.47 (1.30)	0.26	−0.89 (0.87)	0.31	−1.22 (0.71)	0.09
Years lived in USA	0.03 (0.05)	0.55	−0.04 (0.03)	0.19	−0.05 (0.03)	0.09
Education						
Elementary	1.16 (1.62)	0.48	0.75 (1.04)	0.47	1.08 (0.86)	0.21
Some HS	−2.03 (1.21)	0.10	−0.09 (0.81)	0.91	0.65 (0.67)	0.33
Completed HS	−0.92 (1.14)	0.42	−0.45 (0.78)	0.57	0.02 (0.64)	0.98
Associate	−1.84 (1.17)	0.12	−0.07 (0.82)	0.93	0.76 (0.67)	0.26
Bachelor	−1.51 (1.08)	0.16	−0.45 (0.75)	0.55	−0.28 (0.62)	0.65
Masters/Doctoral	Reference		Reference			
Gravidity	−6.47 (2.71)	0.02	−5.69 (1.84)	0.002	−1.82 (1.51)	0.23
Parity	0.30 (0.37)	0.42	0.64 (0.26)	0.01	0.17 (0.21)	0.42
Recently had baby (yes)	0.88 (0.62)	0.16	−0.49 (0.42)	0.24	0.19 (0.35)	0.58
Breastfed previous child (yes)	0.78 (0.73)	0.29	0.92 (0.49)	0.06	1.09 (0.40)	0.01
Close relative breastfed child (yes)	1.55 (0.72)	0.03	2.24 (0.48)	<0.001	1.03 (0.39)	0.01
Constant	23.07 (2.34)	<0.001	19.23 (1.59)	<0.001	17.58 (1.31)	<0.001

*Note*: B=unstandardized beta, SE=standard error. Variance inflation factor values indicated no multicollinearity concerns. Adjusted R square: Attitudes: 0.14, Subjective norms: 0.09, Behavioral control: 0.10.

## Discussion

We found that speakers who preferred Urdu, Uzbek, and Russian had significantly more positive attitudes towards breastfeeding than English speakers. However, language was not significantly associated with any of the TPB variables of subjective norms, perceived behavioural control, and intentions. In our multivariate analyses, we found that increased gravidity was significantly associated with decreased attitudes and subjective norms while increased parity was significantly associated with increased subjective norms. African-Americans had significantly greater subjective norms and approached significance for perceived behavioural control as compared to Whites. However, education level was not significantly associated with breastfeeding attitudes, subjective norms, or perceived behavioural control. Previous breastfeeding experience was associated with increased perceived behavioural control. Having a close relative breastfeed a child was significantly associated with an increase in positive attitudes toward breastfeeding, subjective norms, and perceived behavioural control. The IIFAS and breastfeeding knowledge did not significantly differ among the language groups.

We found that the TPB variable of attitudes towards breastfeeding were more positive among Urdu, Uzbek, and Russian speakers compared to English speakers. Previous research found attitudes were positively associated with exclusive breastfeeding among white and Black participants but not among Hispanics [10]. Our findings for language influencing breastfeeding attitudes are likely based on cultural attitudes among groups speaking these languages. Pakistani women [19] and Russian women [20] are from cultures where traditional gender roles emphasize the mother having more responsibility than the father for caring for children. We speculate that this greater responsibility influences mothers’ interest in breastfeeding their children.

We did not find that language was significantly associated with any of the TPB variables of subjective norms, perceived behavioural control, and intentions. There are mixed findings regarding race/ethnicity and both subjective norms and perceived behavioural control. Subjective norms are positively associated with exclusive breastfeeding among whites and African-Americans, but not among Hispanics [10]. Perceived behavioural control is positively associated with exclusive breastfeeding among Hispanics but not among Whites and African-Americans [10]. Our language findings are similar to the lack of associations seen for certain race/ethnic groups. Not all the factors comprising the TPB may be useful for studying whether a woman's preferred language is associated with breastfeeding.

We found that African-Americans as compared to Whites had significantly greater subjective norms and approached significance for greater perceived behavioural control. African-American mothers historically have lower breastfeeding rates and more negative attitudes towards breastfeeding [21]. Our findings contrast with this pattern and suggest changing attitudes among African-Americans.

We found that increased parity was associated significantly with increased subjective norms towards breastfeeding among different preferred language groups. Previous research reports that parity is positively associated with attitudes and intention towards breastfeeding [22]. Our findings are consistent with this pattern.

Educational level was not associated with parameters of attitudes, subjective norms, or perceived behavioural control regarding breastfeeding. This contrasts with previous data indicating that higher education levels are positively associated with more positive attitudes regarding breastfeeding [23]. We speculate that cultural norms among language speakers may have greater bearing on breastfeeding behaviours than education.

Those who previously breastfed expressed increased perceived behavioural control for breastfeeding. Previous research reports that directed breastfeeding educational interventions increased perceived behavioural control for initiating breastfeeding in women who initially had lower scores in this area [8]. Our findings are similar to this pattern. We suggest that educational programming about breastfeeding tailored to first-time mothers may offer increased perceived behavioural control. This may make breastfeeding more likely to occur.

The IIFAS did not differ among the language groups. The IIFAS is a widely used scale to assess specific attitudes regarding breastfeeding [24,25]. It has been validated in multiple languages. Our general attitudes measure based on the TPB differed among a number of the language groups. It is surprising that a general attitude measure for topics such as bad versus good differed while specific attitudes pertaining to breastfeeding details did not differ among the language groups.

Breastfeeding knowledge did not differ significantly among the language groups. All language groups correctly answered approximately half the questions that probed knowledge. Previous studies on breastfeeding knowledge resulted in diverse results. In some, fewer than half of the questions were answered correctly [26]; in others, most questions were answered correctly [27]. Our findings for breastfeeding knowledge are in between these two patterns. The questions asked may not have been the same, and the level of education that clinics provided to women may have differed. We conclude that many women receive suboptimal education on breastfeeding. We suggest that women should be assessed for their knowledge as part of prenatal care, and then provided with an adequate education if it is lacking.

One strength of this study is the comparison it provides among multiple language groups regarding a number of variables involving breastfeeding. A limitation of the study is that we did not assess the level of traditional gender roles. Future research should include measuring traditional gender roles and their association with breastfeeding topics. Second, we did not study the association of intentions with actual initiation of breastfeeding.

## Conclusion

In conclusion, we found that Urdu, Uzbek, and Russian speakers had significantly more positive attitudes towards breastfeeding than did English speakers. Spanish and English language speakers did not differ in their attitudes towards breastfeeding. To the extent that preferred language is a proxy for culture, clinicians can use this parameter as a basis for directing approaches to lactation education.

### Key Points

- Urdu, Uzbek, and Russian speakers had significantly more positive attitudes towards breastfeeding than did English speakers.
- Spanish and English language speakers did not differ significantly in their attitudes towards breastfeeding.
- To the extent that preferred language is a proxy for culture, clinicians can use this parameter as a basis for directing approaches to lactation education.
